# Economic evaluation of physical activity interventions for type 2 diabetes management: a systematic review

**DOI:** 10.1093/eurpub/ckac074

**Published:** 2022-08-26

**Authors:** Ana Barbosa, Stephen Whiting, Ding Ding, João Brito, Romeu Mendes

**Affiliations:** EPIUnit—Instituto de Saúde Pública, Universidade do Porto, Porto, Portugal; Laboratório para a Investigação Integrativa e Translacional em Saúde Populacional (ITR), Porto, Portugal; EPIUnit—Instituto de Saúde Pública, Universidade do Porto, Porto, Portugal; Laboratório para a Investigação Integrativa e Translacional em Saúde Populacional (ITR), Porto, Portugal; World Health Organization, Regional Office for Europe, Copenhagen, Denmark; Prevention Research Collaboration, Sydney School of Public Health, The University of Sydney, Camperdown, Australia; Portugal Football School, Portuguese Football Federation, Oeiras, Portugal; EPIUnit—Instituto de Saúde Pública, Universidade do Porto, Porto, Portugal; Laboratório para a Investigação Integrativa e Translacional em Saúde Populacional (ITR), Porto, Portugal; World Health Organization, Regional Office for Europe, Copenhagen, Denmark; Portugal Football School, Portuguese Football Federation, Oeiras, Portugal; ACES Douro I—Marão e Douro Norte, Northern Region Health Administration, Vila Real, Portugal

## Abstract

**Background:**

Economic evaluation of physical activity interventions has become an important area for policymaking considering the high costs attributable to physical inactivity. However, the evidence for such interventions targeting type 2 diabetes control is scarce. Therefore, the present study aimed to synthesize economic evaluation studies of physical activity interventions for type 2 diabetes management.

**Methods:**

A systematic review was conducted using the Preferred Reporting Items for Systematic Reviews and Meta-Analyses 2020 statement (PROSPERO reference number CRD42021231021). An electronic search was performed in PubMed, Web of Science, Cochrane Library and NHS Economic Evaluation Database. Studies were eligible if they included: adults with type 2 diabetes; any physical activity intervention in the community settings; an experimental or quasi-experimental design; and a parameter of economic evaluation [cost analysis of interventions, cost-effectiveness analysis (including cost-utility analysis) and cost-benefit analysis] as an outcome.

**Results:**

Ten studies were included in this review: seven were randomized controlled trials and three were quasi-experimental studies. All studies included direct costs, and four also included indirect costs. Four studies demonstrated that physical activity interventions were cost-saving, six studies showed cost-effectiveness, and two studies reported cost-utility. The estimates varied considerably across the studies with different analytical and methodological approaches.

**Conclusion:**

Overall, this systematic review found that physical activity interventions are a worth investment for type 2 diabetes management. However, comparability across interventions was limited due to heterogeneity in interventions type, design and delivery, which may explain the differences in the economic measures.

## Introduction

Diabetes is a major public health challenge of the century. In the last two decades, the prevalence of diabetes has increased alarmingly worldwide.^1^ In 2021, it was estimated that 537 million adults aged 20–79 years were diagnosed with diabetes.^1^ Obesity and physical inactivity have been associated with the increase in the prevalence of type 2 diabetes (T2D)—which accounts for around 90% of all diabetes cases worldwide.^1^

Physical activity is critical to the prevention and management of T2D.^2^^,^^3^ Studies have shown that physical activity decreases glycated hemoglobin, insulin resistance, fasting blood glucose, body mass index, body fat, blood lipids and blood pressure.^4–6^

Unfortunately, most people living with T2D remain physically inactive,^7^^,^^8^ and are, therefore, missing the opportunities to capitalize on the benefits, such as the reduced risk of cardiovascular events and overall mortality,^9^^,^^10^ and lowered healthcare costs. Recent studies showed that physical inactivity is costly and associated with a considerable disease burden.^11^ In 2013, physical inactivity was estimated to cost the healthcare system $53.8 billion globally, and, T2D to cost $37.6 billion to the healthcare system. Physical inactivity-related deaths also contributed to at least $13.7 billion in productivity losses and were responsible for 13.4 million disability-adjusted life-years worldwide.^12^

The economic evaluation of physical activity interventions plays an important role in informing policymaking and resource allocation, considering the constrained resources and competing priorities. In recent years, the economic evaluation of physical activity interventions has become an increasingly common practice. Evidence shows that some physical activity interventions are very cost-effective, such as various school-based interventions among children and adolescents, interventions using pedometers among adults, fall prevention programs among older people, and mass media campaigns and environmental approaches for the general population.^13^

Among people living with T2D, studies have found a varying degree of cost-effectiveness.^14^^,^^15^ Examples of interventions include intensive hypertension control in individuals with T2D (e.g. angiotensin-converting enzyme inhibitor therapy for intensive hypertension control compared with standard hypertension control); the use of pioglitazone plus metformin, a reportedly cost-saving therapy when compared with rosiglitazone plus metformin; comprehensive foot care to prevent ulcers compared with the usual care; counseling and treatment for smoking cessation compared with no counseling and treatment; intensive glycemic control in persons with newly diagnosed T2D compared with conventional glycemic control.^14^^,^^15^ However, evidence regarding the economic evaluation of physical activity interventions for T2D management remains scarce.

Therefore, we aim to systematically review economic evaluation studies of physical activity interventions for T2D management. Specifically, we aimed to summarize cost analysis of interventions (CAI), cost-effectiveness analysis (CEA) (including cost-utility analyses [CUA]), and cost-benefit analysis (CBA) of physical activity interventions in T2D.

By synthesizing and critically appraising existing economic evaluation studies of physical activity interventions in T2D, this study intends to assist decision makers with resource allocation and intervention selection.^16^

## Methods

This systematic review was conducted following the Preferred Reporting Items for Systematic Reviews and Meta-Analyses (PRISMA) 2020 statement^17^ (Supplementary file S1). The protocol of this systematic review was registered in PROSPERO—reference number CRD42021231021.

### Eligibility criteria

Studies were eligible if participants were adults diagnosed with T2D; included any physical activity intervention in the community settings; included experimental and quasi-experimental studies; outcome measures included a parameter of economic evaluation—CAI, CEA (including CUA) or CBA.

We excluded studies with multicomponent interventions (e.g. combined physical activity and diet), and studies with multimorbidity including T2D but without separate data for T2D.

### Information sources

Electronic searches were conducted in PubMed, Web of Science, Cochrane Library and NHS Economic Evaluation Database.

### Search strategy

For database search, we used the following keywords: (‘physical activity’ OR exercise OR ‘active transport’ OR ‘active mobility’ OR ‘active commuting’ OR ‘active travel’ OR walking OR cycling OR running OR training OR sport*) AND (diabet* OR ‘glycemic control’ OR ‘glycaemic control’ OR ‘glucose control’) AND (cost* OR cost-effectiveness OR cost-utility OR cost-benefit OR ‘economic evaluation’ OR ‘economic analysis’ OR ‘economic assessment’ OR ‘economic impact’). We did not apply language and date limits to the searches.

Detailed search strategy is available in Supplementary file S2.

### Selection process

Two authors (A.B. and R.M.) independently reviewed the search results and screened records retrieved from databases according to predefined steps. First, records were screened based on the information from the title and abstract. Second, potentially relevant articles were retrieved for full-text reading and to determine their eligibility. In case of disagreement, the consensus was reached through discussion.

### Data collection process

Two authors (A.B. and R.M.) independently extracted data from eligible studies. Then, retrieved data were compared and discussed if discrepancies existed. A third author (S.W.) reviewed entered data for accuracy. In case of unclear information, the original studies’ authors were contacted to provide additional clarification.

### Data items

We considered studies eligible if they presented at least one of the following economic evaluation outcomes:

- *Cost analysis of interventions (CAI)*: estimation of the costs of an intervention’s implementation. It can include direct costs (costs of resources used to design and implement an intervention, such as personnel time, facility rent, supplies, and medications), productivity losses (impacts of patient participation in an intervention, such as work time lost or leisure-time lost due to participation in the intervention) and intangible costs (non-ﬁnancial costs, such as pain and suffering, which impose a major burden on individuals).^18^
- *Cost-effectiveness analysis (CEA)*: comparison between the costs and the effectiveness of two or more interventions with effectiveness measured in the same units. The incremental cost-effectiveness ratio (ICER) is used to compare interventions—the difference in costs divided by the difference in health effects. Health effects are frequently measured through quality-adjusted life-years (QALYs) gained, disability-adjusted life-years (DALYs) averted, reduction of glycated hemoglobin, increase in daily steps, reduction in body fat, etc.^19^ Generally, when the effectiveness is measured through QALYs, the term cost-utility analysis (CUA) can be used.^15^
- *Cost-benefit analysis (CBA)*: comparison of the costs (including those of implementing an intervention) and benefits (including those resulting from an intervention, such as medical costs averted, productivity gains and the monetized value of health improvements) of an intervention. The unity of analysis is monetary.^18^

For each study, we summarized the following characteristics: first author, year of publication, country, design, intervention and comparison groups (type, duration, measurement, mode of delivery), study length, setting, condition (e.g. T2D vs multimorbidity of T2D), sample size (including sample size for each group, if available), and participants’ age and sex. In addition, we extracted the following methodological information from the studies: the perspective of the analysis, type of economic evaluation, cost analysis, and health outcomes. Finally, we extracted the key findings and the authors’ interpretation of the economic evaluation.

### Study quality assessment

The assessment of the reporting quality of economic evaluation studies was performed by two independent authors (A.B. and R.M.), using the Consolidated Health Economic Evaluation Reporting Standards (CHEERS) statement.^20^ This statement consists of a 24-item checklist subdivided into six main categories: (i) title and abstract; (ii) introduction; (iii) methods; (iv) results; (v) discussion; and (vi) other.

### Effect measures

CAI usually considers the total cost of the interventions’ implementation.

For CBA, the expected monetary benefits of the intervention are subtracted from the total cost of the interventions’ implementation.

For CEA (including CUA), interventions can be classified as cost-saving (an intervention that generates a similar health outcome with fewer costs than the comparison intervention) or cost-effective, according to the interpretation of the intervention impact in the variable that is used to measure the effectiveness (e.g. reduction in glycated hemoglobin, increase in daily steps, reduction in body fat, reduction in medications prescription). ICER—the difference in costs divided by the difference in health effects—is commonly used to measure cost-effectiveness and cost-utility, and it can be compared with thresholds based on per capita national incomes, benchmark interventions, or league tables.^19^

### Synthesis methods

We conducted a narrative synthesis of included studies.

For the comparison of national estimates from different years and in different currencies, we converted all to purchasing power parity (PPP) international dollars using conversion factors provided by the World Bank,^21^ and considering the cost estimate year that studies provided. If a study did not mention the year used in cost analysis, we assumed the cost was the year of publication.

We did not perform meta-analysis to synthesize the results since we found many sources of heterogeneity across the studies.^22^

## Results

### Study selection

**Figure 1:**
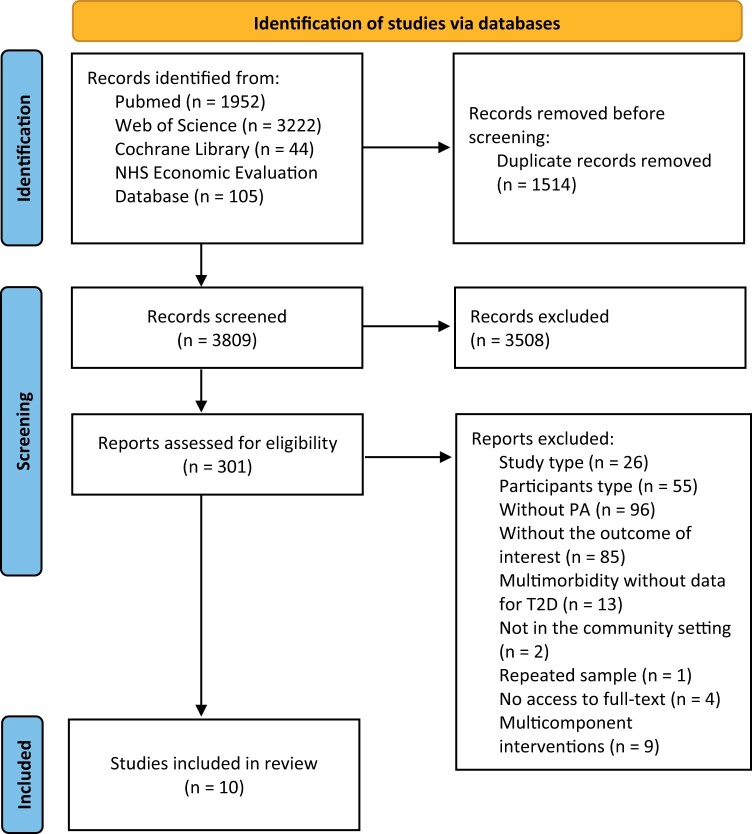
PRISMA 2020 flow diagram. PA, physical activity; T2D, type 2 diabetes

A total of 5323 references were identified in the initial search in electronic databases. After the duplicated studies (*n* = 1514) were removed, 3809 studies remained. After screening for the title and abstract, 3508 papers were excluded, and 301 studies were eligible for full-text reading, from which 291 were removed. Thus, the selection process resulted in the inclusion of 10 studies in the qualitative synthesis (figure 1).

### Study characteristics

The study characteristics included in this review are shown in table 1.

**Table 1:** Characteristics of included studies

Author, year	Country	Study design	Intervention group	Comparison group	Length	Setting	Condition	Sample (*n*)	Age range (mean ± SD), years	Sex
Brun et al., 2008^28^	France	RCT	Exercise program (including brisk walking, jogging or gymnastics)	Usual care	12 months	Community	T2D	25 (13 IG, 12 CG)	40–85 (59.7 ± 2)	M + F(26.0% F)
Coyle et al., 2012^26^	Canada	RCT	Aerobic exercise; resistance exercise; combined exercise	Usual care	6 months	Community	T2D	251	39–70 (54.2)	M + F(34.9% F)
Di Loreto et al., 2005^32^	Italy	Quasi-experimental	Exercise counseling + phone calls + sessions in outpatient clinic	None	24 months	Community	T2D	179	>40 (62 ± 1)	M + F
Johnson et al., 2015^27^	Canada	Quasi-experimental	Pedometer-based walking program	Usual care with a pedometer but without instructions	6 months	Primary Health Care	T2D	186 (94 IG, 92 CG)	≥18 (59.3 ± 8.3)	M + F(50.0% F)
Kaplan 1988^23^	USA	RCT	Exercise; diet; diet + exercise	Education	18 months	Community	T2D	76	53.8 ± 8.0 exercise; 54.9 ± 12.3 diet; 56.9 ± 8.9 diet + exercise; 54.5 ± 8.8 CG	M + F(57.9% F)
Kuo et al., 2021^24^	USA	RCT	Exercise program (EXER); CBT; combined exercise program (EXER) + CBT	Usual care	15 months	Community	T2D with major depressive disorder	140 (EXER 34, CBT 36, EXER + CBT 34, CG 36)	56.0 ± 10.7	M + F(76.0% F)
Marios et al., 2012^29^	Australia	RCT	Walking exercise program monitored by heart rate monitors + phone calls	Walking program	6 months	Community	T2D	26 (15 IG, 11 CG)	18–80 (60.3 ± 9.4 IG, 65.1 ± 7.9 CG)	M + F(33.0% F in IG, 64.0% F in CG)
Pepin et al., 2020^25^	USA	Quasi-experimental	Aerobic + resistance + balance exercise program	None	12 months	Community	Multimorbidity	453	31–91 (67 ± 10)	M + F(6.0% F)
Sultana et al., 2018^30^	Malaysia	RCT	Aerobic exercise; combined (aerobic + resistance/strengthening) exercise program	Usual care	14 weeks	Community; hospital	T2D	75 (25 aerobic training, 25 combined, 25 CG)	40–60	M + F
Taylor et al., 2020^31^	UK	RCT	Exercise referral schemes + e-coachER (a pedometer + fridge magnet with PA recording sheets, and a user guide to access the web-based support in the form of seven ‘steps to health’)	Exercise referral schemes alone	12 months	Community	Multimorbidity	450 (224 IG, 226 CG)	50 ± 13 IG, 51 ± 14 CG	M + F(64.0% F)

CBT, cognitive behavioral therapy; CG, control group; F, female; IG, intervention group; M, male; RCT, randomized controlled trial; SD, standard deviation; T2D, type 2 diabetes.

All studies were published in English, between 1988 and 2021. Half of the studies were conducted in the USA^23–25^ and Canada.^26^^,^^27^ Seven studies were randomized controlled trials (RCT)^23^^,^^24^^,^^26^^,^^28–31^ and three were quasi-experimental studies.^25^^,^^27^^,^^32^ All studies included interventions conducted in the community settings, although one study also included a hospital setting.^30^ The length for included studies varied from 14 weeks to 24 months.

All studies included participants with T2D. Eight studies included only patients with T2D,^23^^,^^24^^,^^26–30^^,^^32^ and two studies had individuals with other chronic conditions but provided data for T2D.^25^^,^^31^ The participants’ age ranged from 18 to 91, and most participants were middle-aged.

Regarding interventions, three were walking programs,^27^^,^^29^^,^^30^ and eight were multicomponent exercise programs (e.g. aerobic, resistance, combined aerobic and resistance exercise programs, etc.).^23–26^^,^^28^^,^^30–32^ Two interventions additionally included phone calls for monitoring weekly minutes of physical activity and counseling.^29^^,^^32^ Two interventions included pedometers^27^^,^^31^ and one intervention included a heart rate monitor^29^ for physical activity self-monitoring.

Most of the comparator groups included participants who followed usual care,^24^^,^^26–28^^,^^30^^,^^31^ one study had education,^23^ one included a walking program only^29^ and one received a pedometer only.^27^

### Study quality assessment in studies

The assessment of methodological quality for each study is presented in Supplementary file S3.

Most of the studies adhered to the CHEERS checklist in the categories of title and abstract, introduction, discussion and other. All the studies did not comply with methods and results’ items, especially the choice of model, assumptions and uncertainty characterization.

### Results of individual studies and synthesis

Results of individual studies are presented in table 2. Four studies took the sole perspective of healthcare,^23^^,^^25^^,^^28^^,^^29^ four studies combined healthcare and societal perspectives^24^^,^^26^^,^^31^^,^^32^ and two studies the payer perspective.^27^^,^^30^

**Table 2:** Results of individual studies

Study	Perspective	Economic evaluation	Cost analysis	Health outcomes	Findings	Authors’ interpretation of the economic evaluation
Brun et al., 2008^28^	Healthcare	CEA	Costs Direct costs: number and duration of hospitalizations, number of outpatient consultations with the family physician or specialists, drugs prescribed and analyses performed. Indirect costs: periods of not working, job loss and unemployment.	Body composition, fitness, metabolic balance, diabetes treatment.	There was no significant change in body composition, blood pressure, lipid profile and glycated hemoglobin in IG, compared with CG; there was a significant reduction (26%) in insulin resistance from 3.39 ± 0.76 to 2.58 ± 0.47 (*P* < 0.05), in the IG, while tended (non-significantly) to increase in the CG (from 2.75 ± 0.59 to 4.34 ± 1.22). Regarding fitness, the intervention prevented loss of maximum aerobic capacity (decreased in the CG, *P* = 0.014), and resulted in a higher maximum power output (*P* = 0.041) and 6-min walking distance (*P* = 0.020). Indirect costs: none. Direct costs: IG required no hospitalizations, the CG spent 1.27 days in hospital (*P* = 0.047) (range: 0–5 days, corresponding to a mean total cost of $1250.25). The total cost of healthcare dropped by 50% in the IG ($1.87 ± 1 per day vs. $3.40 ± 1.67 per day, *P* = 0.018). The difference in disease-related costs after versus before the study was +0.26 per day in the CG versus $0.009 per day in the IG (*P* = 0.002). There was a significant reduction in sulfonyl urea treatments (−13.7 ± 6%, *P* < 0.05) in IG, compared with CG. The IG also reduced metformin, acarbose and insulin treatments.	Intervention is cost-saving.
Coyle et al., 2012^26^	Healthcare; societal	CAI CEA assessed by ICER (the additional costs per QALYs)	Costs: Intervention costs: lifetime membership to a health club and exercise specialist. Direct costs: costs of managing T2D with or without complications.	Life expectancy and quality-adjusted life expectancy.	The combined exercise was the most expensive ($32445.18), followed by the aerobic exercise ($31797.08), the resistance exercise ($31027.47) and no exercise program ($25174.38). Both life expectancy and quality-adjusted life expectancy were highest for the combined exercise (life-years = 11.79, QALYs = 8.94) compared with aerobic exercise (life-years = 11.57, QALYs = 8.77), resistance exercise (life-years = 11.51, QALYs = 8.73), and no exercise program (life-years = 11.48, QALYs = 8.70). The ICER was $167682.01 per QALYs, $94615.96 per QALYs, and $30680.74 per QALYs for the resistance, aerobic and combined exercise programs, respectively, compared with no exercise program. The ICER for the combined program was $3882.08 per QALYs compared with aerobic exercise, and $6942.70 per QALYs compared with the resistance exercise. The combined exercise resulted in the greatest increase in life expectancy and quality-adjusted life expectancy. At a maximum value of $61.72 per QALYs, the combined exercise remained cost-effective compared with the three alternatives.	The combined exercise program is more cost-effective than aerobic, resistance or no exercise program in the improvement of T2D control
Di Loreto et al., 2005^32^	Healthcare; societal	CEA	Costs Direct costs: expenses for medications and other costs usually paid by the National Health Service (e.g. counseling intervention, laboratory testing, hospitalization and outpatient care). Direct social costs include the value of participants' time spent in practicing exercise, cost of related items (shoes, fitness equipment, etc.), transportation to exercise places and admission to health clubs. Indirect social costs: include the time that participants reported as lost from work or usual activities as a result of counseling visits, illness or injury; each day lost to morbidity was valued at $100	Improvement in the 10-year coronary heart disease risk, glycemic control and cardiovascular risk factors, reduction in medical and social costs.	There were significant (*P* < 0.0001) reductions in body weight, body mass index, waist circumference, fasting plasma glucose, glycated hemoglobin, systolic and diastolic blood pressure, heart rate, total and LDL cholesterol, and triglycerides; and 3% reduction in 10-year coronary heart disease risk; there was a significant (*P* < 0.0001) increase in HDL cholesterol. Improvements in glycemic control and cardiovascular risk factors were associated with significant (*P* < 0.0001) reductions in medical and social costs, for a total saving of $855 per capita per year. METs per hour per week were inversely related with medical prescription costs (*r* = 0.51, $18), other medical costs (*r* = 0.33, $23), indirect social costs (*r* = 0.40, $36) and total costs (*r* = 0.60, $66), and positively related with direct social costs (*r* = 0.44, $13), *P* < 0.0001. A 3-mile daily walk was estimated to reduce medication costs by $550, other medical costs by $700, indirect social costs by $1100 and total costs by $2000, and to increase direct social costs by $400. After 24-month, the number of subjects on insulin therapy fell by 25% (before 59/179, after 44/179, *P*â= 0.0003). There was a significant (*P*â< 0.0001) inverse correlation between METs per hour per week and daily units of insulin (*r* = 0.38, 0.35 IU/day).	Intervention is cost-saving.
Johnson et al., 2015^27^	Payer	CAI CEA assessed by ICER (the additional cost per 1000 additional steps achieved).	Costs Intervention costs: included activity time and training of the exercise specialists, administrative support personnel, recreation facilities, supplies, equipment and primary care networks’ overhead. Healthcare costs (direct costs): physician services, hospital outpatient visits and hospital inpatient admissions.	Change in daily steps.	The total costs of the intervention were $274.21 per participant. IG incurred less cost in all categories (physician, out- and inpatient costs) than the CG during the follow-up period. The difference in total costs between-groups was $82.26 per participant. Daily steps increased for the IG compared with CG at 3 months (1292 vs. 418) and 6 months (1481 vs. 336), *P* = 0.002). IG had an incremental rate of 919 steps, compared with an unadjusted increment of 393 steps in CG, *P* < 0.001. The ICER was $89.52 per 1000 daily steps.	Intervention is cost-effective in increasing number of daily steps.
Kaplan, 1988^23^	Healthcare	CAI CUA measured as the additional cost per well-year.	Costs Direct costs: history and physical examination, laboratory charges, ECG evaluations, charges for behavior modification sessions, and charges for medical consultation.	Quality of well-being.	The total costs of the program were approximately $1000 per participant. The diet + exercise group showed improvement in quality of well-being throughout the study, with a decrease at 12 months. The diet and exercise groups had experienced 0.06 units of improvement on the quality of well-being at 18 months, compared with −0.04 for the CG (difference equal to 0.092 quality of well-being units, *P* < 0.01). The cost-utility ratio was $10 870 per well-year.	The diet and exercise groups have cost-utility, compared with other behavioral programs.
Kuo et al., 2021^24^	Healthcare; societal	CAI CEA assessed by ICER (additional costs per QALY).	Costs Intervention costs: implementing the interventions by CBT therapists and exercise trainers, passes/memberships to fitness facilities, participant time spent participating in sessions Medical costs (outpatient visits, emergency care and room, hospitalization services; laboratory testing; and self-monitoring of blood glucose); Informal healthcare costs: time costs of informal caregivers in caring for patients, or transportation costs.	Incidence of clinical outcomes (e.g. stroke, cardiovascular death, myocardial infarction), life expectancy and quality-adjusted life expectancy.	Per participant, intervention-related costs over 15 months were $1615, $1532, $1983 and $2138 for the CG, exercise program, CBT and exercise program + CBT groups, respectively. Over a 10-year period, the exercise program + CBT was associated with the longest quality-adjusted life expectancy (5.355 QALYs), followed by exercise program (5.047), CBT (4.955) and CG (4.665). The exercise program resulted in the lowest total costs over 10 years ($75714). Healthcare perspective: compared with CG, the exercise program strategy saved $313 per patient and produced 0.382 more QALYs and the exercise program + CBT saved $403 more and gained 0.690 more QALY (ICER of $600 per QALY). Compared with exercise program, exercise program + CBT cost $716 more and gained 0.308 more QALY, (ICER of around $2300 per QALY gained). Societal perspective: the exercise program strategy saved $126. Compared with CG, the ICER of exercise program and exercise program + CBT was around $800 and $2000 per QALY gained. Compared with exercise program, the ICER for exercise program + CBT was around $3500 per QALY.	Exercise program and exercise program + CBT interventions are cost-saving; exercise program + CBT is more cost-effective than exercise program or CBT alone.
Marios et al., 2012^29^	Healthcare	CAI CEA	Costs Intervention costs: included heart rate monitors, exercise test consumables, physician supervision of exercise tests and salary for exercise physiologist.	Exercise adherence (number of hours of exercise completed), improvements in peak maximal oxygen uptake, glycated hemoglobin and quality of life; cost-effectiveness of exercise training compared with pharmacological therapy.	The total cost of administering the telemonitored exercise program was $27 300, or $1050 per patient. Costs per patient are similar to the costs that would be borne by the patient for using low dose insulin ($800) and a blood pressure agent ($130) for 6 months. IG completed a mean weekly volume of 138 min, moderate intensity exercise, while CG patients completed 58 minutes weekly (*P* < 0.02). In the IG, peak of maximal oxygen uptake increased (5.5%), and treadmill time (18%) and maximum heart rate (3%) were significantly greater at 6 months, compared with CG (*P* = 0.04). Glycated hemoglobin did not change significantly after 6 months (*P* = 0.46). No significant between-group changes were seen for quality of life.	The amount invested in intervention is comparable with other health interventions and it improved some health outcomes.
Pepin et al., 2020^25^	Healthcare	CEA	Costs Direct costs: included the costs of medication. Net changes over 12 months in cost were calculated by subtracting the cost related to increases from the cost related to decreases in fills and associated costs for the prescriptions fills.	Changes in medication use and cost of medication classes commonly prescribed in the management of chronic conditions.	After 12 months, participants reduced the number of active prescriptions by 25%, 65% had no change and 10% increased diabetes medication, a net change of 14% decrease in diabetes medications. Fifty-five percent of patients had a decrease in their overall number of fills, with an average associated cost decrease of $473 per fill, or $117.254 overall. A net reduction was found in diabetes medications ($2.212).	Intervention is cost-saving.
Sultana et al., 2018^30^	Payer	CAI CEA assessed by ICER (the additional cost per health status)	Costs Direct costs: included the costs related to program implementation and running.	Glycated hemoglobin and health status.	Direct costs: $2193.71 and $2147.60 for combined and aerobic exercise interventions, respectively. There were significant improvements in glycated hemoglobin between aerobic exercise versus CG, and combined exercise versus CG, *P* < 0.001. There were significant improvements in health status between aerobic exercise versus combined exercise (*P* = 0.003), and combined exercise versus CG (*P* < 0.001). The ICER of combined exercise was $5.56 per health status; ICER of aerobic exercise intervention was $827.03 per health status.	Combined exercise program is more cost-effective than aerobic exercise or CG.
Taylor et al., 2020^31^	Healthcare; societal	CAI CUA measured as the additional cost per QALYs CEA assessed by ICER (the additional cost per change in moderate and vigorous PA minutes).	Costs (direct and indirect): Intervention costs: set-up and design of the intervention; delivery of the intervention including handbooks, pedometers, the guide for using the LifeGuide platform, technical support and maintenance of the website; consultation provided by an exercise specialist and staff support to participants Direct and .indirect costs: primary and secondary health service use, prescriptions, hospital admissions, accident and emergency visits; time and money expenses borne by participants about participation in the intervention (e.g. time spent on web platform), visit to exercise specialist and PA.	Quality of life, and minutes of moderate and vigorous PA in ≥10-min bouts.	The average cost per participant was $1970.30 and $2607.19 in the CG and IG, respectively, not statistically significant. The IG (mean 0.662, 95% CI 0.625–0.701) had more QALYs than the CG (mean 0.637, 95% CI 0.585–0.688). The difference in QALYs (0.026, 95% CI 0.013–0.040) between groups was statistically significant. Compared with the CG, the IG cost an additional $24552.36 per QALYs. IG had a weak indicative effect on total weekly minutes of moderate and vigorous PA in bouts of ≥10 min (mean difference 11.8 min, 95% CI –2.1 to 26.0 min), compared with CG. Compared with the CG, the IG cost $25.58 per additional minute of moderate and vigorous PA in a bout of ≥10 min. Compared with the base-case findings, subgroup analysis showed the intervention to be more cost-effective in groups that reported that T2D was the primary reason for referral (ICER $21645.62 per QALYs).	Cost-utility of IG compared with CG in increasing the quality of life. Cost-effectiveness of IG compared with CG in increasing minutes of moderate and vigorous PA ≥ 10-min bouts.

CAI, cost analysis of interventions; CBT, cognitive behavioral therapy; CEA, cost-effectiveness analysis; CG, comparator group; CUA, cost-utility analysis; IG, intervention group; MET, metabolic equivalent of task; PA, physical activity; QALYs, quality-adjusted life years; T2D, type 2 diabetes.

All studies included estimates of direct healthcare costs of physical activity interventions. Direct costs included medical costs (outpatient care, laboratory testing, expenses with medications, hospitalizations and emergency care). Four studies also provided indirect costs,^24^^,^^28^^,^^31^^,^^32^ and included periods of not working, job loss and unemployment, and participants’ time spent in physical activity sessions.

Regarding economic evaluation, nine studies included CEA,^24–32^ and two studies used CUA.^23^^,^^31^ No CBA was identified in this review.

Physical activity interventions were reported as being cost-saving in four studies,^24^^,^^25^^,^^28^^,^^32^ six were considered cost-effective^24^^,^^26^^,^^27^^,^^29–31^ and two with cost-utility (table 3).^23^^,^^31^

**Table 3:** Summary of result of the economic evaluation in individual studies

Study	Result of the economic evaluation
Brun et al., 2008^28^	Cost-saving
Coyle et al., 2012^26^	Cost-effectiveness
Di Loreto et al., 2005^32^	Cost-saving
Johnson et al., 2015^27^	Cost-effectiveness
Kaplan, 1988^23^	Cost-utility
Kuo et al., 2021^24^	Cost-saving
	Cost-effectiveness
Marios et al., 2012^29^	Cost-effectiveness
Pepin et al., 2020^25^	Cost-saving
Sultana et al., 2018^30^	Cost-effectiveness
Taylor et al., 2020^31^	Cost-utility
	Cost-effectiveness

## Discussion

This systematic review summarized the available evidence on economic evaluation studies of physical activity interventions in the context of T2D control. Of the 10 studies included, nine were conducted in high-income countries, and only one was in a middle-income country (Malaysia).^30^ Overall, we found that physical activity interventions are a worth investment for T2D control. Four interventions were considered cost-saving,^24^^,^^25^^,^^28^^,^^32^ six were considered cost-effective^24^^,^^26^^,^^27^^,^^29–31^ and two were considered to have cost-utility.^23^^,^^31^

In the literature, we can find other examples of interventions that are cost-effective in T2D management. Examples include interventions for hypertension control (angiotensin-converting enzyme inhibitor therapy for intensive compared with standard hypertension control); multicomponent interventions for diabetic risk factors control and early detection of complications, compared with standard glycemic control for persons with T2D; intensive lifestyle interventions to prevent T2D among persons with impaired glucose tolerance, compared with standard lifestyle recommendations; annual screening for diabetic retinopathy and ensuing treatment in persons with T2D, compared with no screening.^14^^,^^15^

Despite the encouraging findings on the investment of physical activity interventions for T2D control highlighted in the current review, it should be noted that only four studies used glycated hemoglobin as the endpoint of T2D control.^28–30^^,^^32^ Other studies used endpoints such as a change in daily steps^27^ or a change in exercise volume^29^^,^^31^ that can indirectly produce health benefits in people with T2D. However, these changes may not be sufficient to improve glycated hemoglobin, as noted by Marios et al.^29^

Consistent in this review, combined aerobic and resistance exercise programs showed cost-effectiveness when compared with the usual care.^26^^,^^30^ These results align with the physical activity guidelines for individuals with T2D, which recommend the inclusion of aerobic, resistance, flexibility and balance exercise training.^2^^,^^3^ These findings also encourage decision makers to allocate resources to multi-modal physical activity interventions.

The reported costs of three interventions were around $1000 per participant,^23^^,^^27^^,^^29^ which is a promising finding since it is similar to the annual per capita expenditure ($1423 in 2012 US dollars) on prescription medications for persons with T2D in the USA.^33^ Additionally, costs are likely to decrease over time with, for example, the reduction of the prescribed medication for T2D and cardiovascular risk factors.^25^^,^^32^ Moreover, regular practice of physical activity contributes to preventing and managing cognitive and mental health conditions and improving physical function across the lifespan.^34^^,^^35^

In this review, most studies took a healthcare perspective.^23–26^^,^^28^^,^^29^^,^^31^^,^^32^ Despite being very relevant for policymakers, upcoming studies should consider the societal perspective, which incorporates the economic impact on society, including the health sector (direct costs to public and private healthcare systems), non-health sector (indirect costs or productivity losses) and households (impact on usual activities).^36^ By taking a societal perspective, the economic evaluation will be relevant to stakeholders from different sectors, considering that physical activity interventions may involve non-health sectors and the costs of physical inactivity are borne by different sectors of the society.

This study has several limitations that need to be addressed. First, high heterogeneity in interventions type, design and delivery was noted in the included studies, which may explain the differences in the economic measures. Second, similar to a previous systematic review of physical activity-related cost-of-illness analysis, important information, such as time horizon, assumptions, sensitivity analyses, discounting, uncertainty, economic perspective, physical activity and economic measures calculation, was often not reported.^11^ Incomplete reporting has further complicated comparison between studies. Reporting of future studies should follow the best-practice standards for economic evaluation studies, such as the CHEERS statement.^20^ Moreover, recruitment costs were not considered in the majority of the studies, which may have resulted in the underestimation of intervention costs, which is an important point of consideration if programs are scaled up. Third, we did not include multicomponent interventions (such as diet plus exercise), which seem to be more effective and cost-effective for T2D control.^37^

Future research should involve larger studies with robust designs to establish the effects of physical activity interventions on T2D management and its cost-effectiveness, increase the reliability of findings and, ultimately, promote their use by policymakers.

It would also be important to investigate the impact of physical activity interventions on long-term outcomes related to T2D, namely in the incidence of cardiovascular diseases, micro- and macrovascular complications, years of life lost, and mortality. Furthermore, it was possible to note that long-term follow-up studies tend to be more cost-effective given that health benefits often last beyond the study period.

## Conclusion

In conclusion, this systematic review found that physical activity interventions are a worth investment for type 2 diabetes management. However, due to the studies’ heterogeneity, it is challenging to compare interventions across studies.

Studies with a societal perspective and robust analysis over wider time horizons are needed to explore the potential of physical activity interventions in the effectiveness and cost-effectiveness of T2D management over the long term. This will allow for efficient resource allocation by policymakers across the sectors involved in implementation programs.

## Supplementary data

Supplementary data are available at *EURPUB* online.

## Funding

This work was funded by the Portuguese Foundation for Science and Technology within the scope of projects UIDB/04750/2020 and LA/P/0064/2020. Ana Barbosa was supported by the Portuguese Foundation for Science and Technology, grant number SFRH/BD/136702/2018.

## Disclaimer

S.W. is a WHO staff member and R.M. is a WHO consultant. The authors alone are responsible for the views expressed in this publication. They do not necessarily represent the views, decisions, or policies of the institutions they are affiliated with.

*Conflicts of interest*: none declared.

Key points

- Physical activity interventions are a good investment for type 2 diabetes management.
- Health benefits of physical activity interventions last beyond the intervention period.
- Future studies should include a societal perspective, robust design and accurate reporting to better inform resource allocation and decision making.

## Data availability

The data underlying this article will be shared on reasonable request to the corresponding author.

## Supplementary Material

ckac074_Supplementary_Data
