# Supplementary material for: Economic evaluation of physical activity interventions for type 2 diabetes management: a systematic review
Source: Eur J Public Health. 2022 Aug 26;32(Suppl 1):i56–66. doi: 10.1093/eurpub/ckac074 (PMC9421413; doi:10.1093/eurpub/ckac074)
Supplement: ckac074_Supplementary_Data [file ckac074_supplementary_data.zip › Supplementary file 2 (2).pdf]

Economic evaluation of physical activity interventions for type 2 diabetes control: a systematic review

Supplementary file 2 - Full search strategy

PubMed
(“physical activity” OR exercise OR “active transport” OR “active mobility” OR “active commuting” OR “active travel” OR walking OR cycling OR running OR “swimming” OR “aquatic exercise” OR training OR sport*)
AND (diabet* OR “glycemic control” OR “glycaemic control” OR “glucose control”)
AND (cost* OR cost-effectiveness OR cost-utility OR cost-benefit OR “economic evaluation” OR “economic analysis” OR “economic assessment” OR “economic impact”)
Title/Abstract
Results: 1952

Web of Science
(“physical activity” OR exercise OR “active transport” OR “active mobility” OR “active commuting” OR “active travel” OR walking OR cycling OR running OR “swimming” OR “aquatic exercise” OR training OR sport*)
AND (diabet* OR “glycemic control” OR “glycaemic control” OR “glucose control”)
AND (cost* OR cost-effectiveness OR cost-utility OR cost-benefit OR “economic evaluation” OR “economic analysis” OR “economic assessment” OR “economic impact”)
Topic
Results: 3222

Cochrane Library
(“physical activity” OR exercise OR “active transport” OR “active mobility” OR “active commuting” OR “active travel” OR walking OR cycling OR running OR “swimming” OR “aquatic exercise” OR training OR sport*)
AND (diabet* OR “glycemic control” OR “glycaemic control” OR “glucose control”)
AND (cost* OR cost-effectiveness OR cost-utility OR cost-benefit OR “economic evaluation” OR “economic analysis” OR “economic assessment” OR “economic impact”)
Title Abstract Keyword - in Cochrane Reviews (Word variations have been searched)
Results: 44

NHS Economic Evaluation Database
(exercise) AND (diabetes) AND (economic evaluation)
Any field
Results: 105
