# Supplementary material for: Economic evaluation of physical activity interventions for type 2 diabetes management: a systematic review
Source: Eur J Public Health. 2022 Aug 26;32(Suppl 1):i56–66. doi: 10.1093/eurpub/ckac074 (PMC9421413; doi:10.1093/eurpub/ckac074)
Supplement: ckac074_Supplementary_Data [file ckac074_supplementary_data.zip › Supplementary file 3 (1).pdf]

Economic evaluation of physical activity interventions for type 2 diabetes control: a systematic review

Supplementary file 3 - Study quality assessment using the Consolidated Health Economic Evaluation Reporting Standards statement

Item/ Study	1	2	3	4	5	6	7	8	9	10	11	12	13	14	15	16	17	18	19	20	21	22	23	24	% met
Brun et al, 2008 [28]	Yes	Yes	Yes	Yes	Yes	Yes	Yes	Yes	No	Yes	Yes	Yes	Yes	Yes	No	No	No	Yes	Yes	No	Yes	No	Yes	No	70.8
Coyle et al, 2012 [26]	Yes	Yes	Yes	Yes	Yes	Yes	Yes	Yes	Yes	Yes	Yes	Yes	Yes	Yes	Yes	Yes	Yes	Yes	Yes	Yes	Yes	Yes	Yes	Yes	100
Di Loreto et al, 2005 [32]	No	Yes	Yes	Yes	No	Yes	No	Yes	No	Yes	Yes	Yes	Yes	Yes	No	No	Yes	Yes	Yes	No	No	Yes	No	No	58.3
Johnson et al, 2015 [27]	Yes	Yes	Yes	Yes	Yes	Yes	Yes	Yes	Yes	Yes	Yes	Yes	Yes	Yes	Yes	No	Yes	Yes	Yes	Yes	Yes	Yes	Yes	Yes	95.8
Kaplan, 1988 [23]	Yes	Yes	No	No	Yes	Yes	Yes	Yes	Yes	Yes	No	Yes	Yes	Yes	Yes	Yes	No	Yes	Yes	No	Yes	Yes	Yes	No	75.0
Kuo et al, 2021 [24]	Yes	Yes	Yes	Yes	Yes	Yes	Yes	Yes	Yes	Yes	Yes	Yes	Yes	Yes	Yes	Yes	Yes	Yes	Yes	Yes	Yes	Yes	Yes	Yes	100
Marios et al, 2012 [29]	No	Yes	Yes	Yes	Yes	Yes	Yes	Yes	No	Yes	Yes	Yes	No	No	No	No	No	Yes	Yes	No	No	Yes	Yes	No	58.3
Pepin et al, 2020 [25]	Yes	Yes	No	No	Yes	No	No	Yes	No	Yes	No	Yes	No	No	No	No	No	Yes	Yes	No	No	Yes	Yes	Yes	45.8
Sultana et al, 2018 [30]	Yes	Yes	Yes	Yes	Yes	Yes	Yes	Yes	No	Yes	No	Yes	Yes	Yes	Yes	Yes	No	Yes	Yes	No	Yes	No	Yes	No	75.0
Taylor et al, 2020 [31]	No	Yes	Yes	Yes	Yes	Yes	Yes	Yes	Yes	Yes	Yes	Yes	Yes	Yes	No	No	Yes	Yes	Yes	Yes	Yes	Yes	Yes	Yes	87.5
